# Dimethyl­ammonium guanidinium naphthalene-1,5-disulfonate

**DOI:** 10.1107/S1600536812011099

**Published:** 2012-03-21

**Authors:** Bin Wei

**Affiliations:** a Ordered Matter Science Research Center, Southeast University, Nanjing 211189, People’s Republic of China

## Abstract

The asymmetric unit of the title salt, CH_6_N_3_^+^·C_2_H_8_N^+^·C_10_H_6_O_6_S_2_^2−^, consists of one dimethyl­ammonium cation, one guanidinium cation, and two half naphthalene-1,5-disulfonate anions, which lie on inversion centers. N—H⋯O hydrogen bonds link the cations and anions into layers parallel to the *ab* plane. The layers have a sandwich-like structure, with the sulfonate groups and cations forming outer slices and the naphthalene ring systems inside.

## Related literature

For nanoporous materials with two-dimensional hydrogen-bonded networks, see: Russell *et al.* (1997 ▶). For recent studies of organic and organic–inorganic salts with ferroelectric properties, see: Fu *et al.* (2009 ▶); Wu *et al.* (2011 ▶). For general background to structure phase transitions in closely related compounds, see: Ye *et al.* (2009 ▶); Zhang *et al.* (2010 ▶).
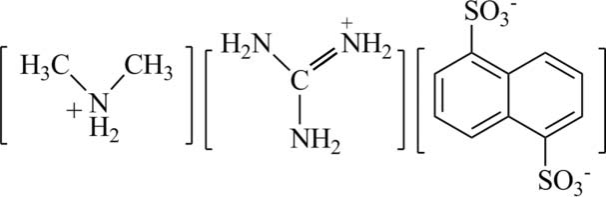


## Experimental

### 

#### Crystal data

CH_6_N_3_^+^·C_2_H_8_N^+^·C_10_H_6_O_6_S_2_^2−^
*M* *_r_* = 392.45
Triclinic, 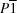

*a* = 8.7782 (18) Å
*b* = 9.0316 (18) Å
*c* = 11.923 (2) Å
α = 87.10 (3)°
β = 74.74 (3)°
γ = 88.77 (3)°
*V* = 910.7 (3) Å^3^
*Z* = 2
Mo *K*α radiation
μ = 0.33 mm^−1^
*T* = 293 K
0.20 × 0.20 × 0.20 mm

#### Data collection

Rigaku SCXmini diffractometer
Absorption correction: multi-scan (*CrystalClear*; Rigaku, 2005 ▶) *T*_min_ = 0.936, *T*_max_ = 0.937
9502 measured reflections
4168 independent reflections
3097 reflections with *I* > 2σ(*I*)
*R*_int_ = 0.031

#### Refinement

*R*[*F* ^2^ > 2σ(*F* ^2^)] = 0.046
*wR*(*F* ^2^) = 0.121
*S* = 1.04
4168 reflections
228 parameters
H-atom parameters constrained
Δρ_max_ = 0.23 e Å^−3^
Δρ_min_ = −0.29 e Å^−3^

### 

Data collection: *CrystalClear* (Rigaku, 2005 ▶); cell refinement: *CrystalClear*; data reduction: *CrystalClear*; program(s) used to solve structure: *SHELXTL* (Sheldrick, 2008 ▶); program(s) used to refine structure: *SHELXTL*; molecular graphics: *SHELXTL*; software used to prepare material for publication: *SHELXTL*.

## Supplementary Material

Crystal structure: contains datablock(s) I, global. DOI: 10.1107/S1600536812011099/yk2047sup1.cif

Structure factors: contains datablock(s) I. DOI: 10.1107/S1600536812011099/yk2047Isup2.hkl

Supplementary material file. DOI: 10.1107/S1600536812011099/yk2047Isup3.cml

Additional supplementary materials:  crystallographic information; 3D view; checkCIF report

## Figures and Tables

**Table 1:** Hydrogen-bond geometry (Å, °)

*D*—H⋯*A*	*D*—H	H⋯*A*	*D*⋯*A*	*D*—H⋯*A*
N1—H1*D*⋯O1^i^	0.86	2.10	2.916 (3)	159
N1—H1*E*⋯O5	0.86	2.02	2.825 (3)	157
N2—H2*D*⋯O6^ii^	0.86	2.12	2.942 (3)	160
N2—H2*E*⋯O2	0.86	2.08	2.921 (3)	164
N3—H3*A*⋯O4	0.86	2.24	3.084 (3)	167
N3—H3*B*⋯O3	0.86	2.11	2.940 (3)	163
N4—H4*A*⋯O6^iii^	0.90	2.12	3.011 (3)	168
N4—H4*A*⋯O5^iii^	0.90	2.50	3.133 (3)	128
N4—H4*B*⋯O1^iv^	0.90	2.60	3.152 (3)	121
N4—H4*B*⋯O2^iv^	0.90	2.04	2.914 (3)	163

Symmetry codes: (i) 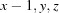; (ii) 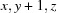; (iii) 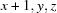; (iv) 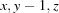.

**Fig. 1.**
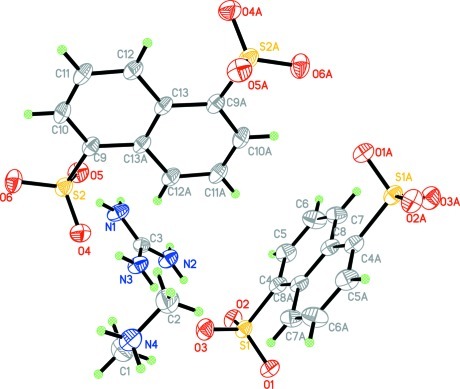
Asymmetric unit of the title compound, with the atomic numbering scheme. Displacement ellipsoids are drawn at the 30% probability level.

**Fig. 2.**
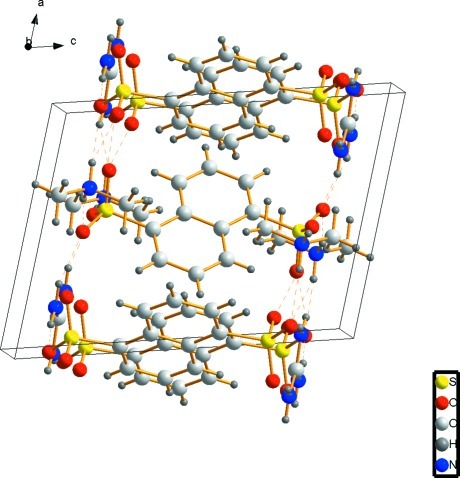
A view of the packing of the title compound. Dashed lines indicate hydrogen bonds.

## supplementary crystallographic information

### Comment

Recently a series of nanoporous materials has been reported, which have two-dimensional hydrogen-bond networks and adjustable porosity (Russell *et al.*, 1997). Guanidinium ions and the sulfonate groups of arenedisulfonate ions can form rich variety of H-bonds. We prepared the title compound in attempts to find new hydrogen-bonded dielectric materials consisting of guanidinium and naphthalene-1,5-disulfonate ions. Unfortunately, the study of dielectric permeability of the title compound indicated that its dielectric constant is essentially temperature-independent below its melting point (388 — 390 K). Thus we have found that the title compound has no dielectric disuniform from 80 K to 405 K.

At room temperature (25°C), the asymmetric unit of the title compound consists of one dimethylammonium cation, one guanidinium cation, and two halves of naphthalene-1,5-disulfonate anions, which lie at inversion centers (Fig. 1). The N—H···O hydrogen bonds join cations and anions into layers parallel to the *ab* plane. Layers have sandwich-like structure: sulfonate groups and cations form outer slices and naphthalene bicycles are inside. (Fig. 2).

### Experimental

The 1,5-naphthalenedisulfonic acid (1.824 g 8 mmol) and guanidinium tetrafluoroborate (0.588 g 4 mmol) were combined in 30 ml aqueous solution, and methanol solution of dimethylamine (0.326 g 4 mmol) was added to the mixture. The solution was stirred for 30 min to complete the reaction, and good quality blocky single crystals were obtained by slow evaporation of the filtrate after two weeks (chemical yield is 62%).

### Refinement

All H atoms were placed in geometrically idealized positions and constrained to ride on their parent atoms with C—H = 0.91—0.93 Å, N—H = 0.86 Å and with *U*_iso_(H) = 1.2 *U*_iso_(C, N) or 1.5 *U*_iso_(C) for methyl H atoms.

### Crystal data

**Table:**

CH_6_N_3_^+^·C_2_H_8_N^+^·C_10_H_6_O_6_S_2_^2^^−^	*Z* = 2
*M**_r_* = 392.45	*F*(000) = 412
Triclinic, *P*1	*D*_x_ = 1.431 Mg m^−^^3^
Hall symbol: -P 1	Mo *K*α radiation, λ = 0.71073 Å
*a* = 8.7782 (18) Å	Cell parameters from 3638 reflections
*b* = 9.0316 (18) Å	θ = 3.0–27.5°
*c* = 11.923 (2) Å	µ = 0.33 mm^−^^1^
α = 87.10 (3)°	*T* = 293 K
β = 74.74 (3)°	Block, colourless
γ = 88.77 (3)°	0.20 × 0.20 × 0.20 mm
*V* = 910.7 (3) Å^3^	

### Data collection

**Table:**

Rigaku SCXmini diffractometer	4168 independent reflections
Radiation source: fine-focus sealed tube	3097 reflections with *I* > 2σ(*I*)
Graphite monochromator	*R*_int_ = 0.031
ω scans	θ_max_ = 27.5°, θ_min_ = 3.3°
Absorption correction: multi-scan (*CrystalClear*; Rigaku, 2005)	*h* = −11→11
*T*_min_ = 0.936, *T*_max_ = 0.937	*k* = −11→11
9502 measured reflections	*l* = −15→15

### Refinement

**Table:**

Refinement on *F*^2^	Primary atom site location: structure-invariant direct methods
Least-squares matrix: full	Secondary atom site location: difference Fourier map
*R*[*F*^2^ > 2σ(*F*^2^)] = 0.046	Hydrogen site location: inferred from neighbouring sites
*wR*(*F*^2^) = 0.121	H-atom parameters constrained
*S* = 1.04	*w* = 1/[σ^2^(*F*_o_^2^) + (0.0492*P*)^2^ + 0.289*P*] where *P* = (*F*_o_^2^ + 2*F*_c_^2^)/3
4168 reflections	(Δ/σ)_max_ < 0.001
228 parameters	Δρ_max_ = 0.23 e Å^−^^3^
0 restraints	Δρ_min_ = −0.29 e Å^−^^3^

### Special details

**Table:**

Geometry. All s.u.'s (except the s.u. in the dihedral angle between two l.s. planes) are estimated using the full covariance matrix. The cell s.u.'s are taken into account individually in the estimation of s.u.'s in distances, angles and torsion angles; correlations between s.u.'s in cell parameters are only used when they are defined by crystal symmetry. An approximate (isotropic) treatment of cell s.u.'s is used for estimating s.u.'s involving l.s. planes.
Refinement. Refinement of *F*^2^ against ALL reflections. The weighted *R*-factor *wR* and goodness of fit *S* are based on *F*^2^, conventional *R*-factors *R* are based on *F*, with *F* set to zero for negative *F*^2^. The threshold expression of *F*^2^ > σ(*F*^2^) is used only for calculating *R*-factors(gt) *etc*. and is not relevant to the choice of reflections for refinement. *R*-factors based on *F*^2^ are statistically about twice as large as those based on *F*, and *R*- factors based on ALL data will be even larger.

### Fractional atomic coordinates and isotropic or equivalent isotropic displacement parameters (Å^2^)

**Table:**

	*x*	*y*	*z*	*U*_iso_*/*U*_eq_	
S1	0.56454 (6)	0.60040 (6)	0.20852 (5)	0.04580 (17)	
O1	0.72470 (18)	0.64764 (18)	0.19535 (15)	0.0537 (4)	
O2	0.47542 (19)	0.71224 (18)	0.15867 (15)	0.0554 (4)	
O3	0.5537 (2)	0.45431 (18)	0.16728 (16)	0.0605 (5)	
C4	0.4733 (2)	0.5921 (2)	0.3610 (2)	0.0444 (5)	
C5	0.3440 (3)	0.6794 (3)	0.4035 (2)	0.0574 (6)	
H5	0.3017	0.7376	0.3523	0.069*	
C6	0.2749 (3)	0.6819 (3)	0.5227 (3)	0.0650 (7)	
H6	0.1878	0.7430	0.5500	0.078*	
C7	0.3320 (3)	0.5973 (3)	0.5998 (2)	0.0523 (6)	
H7	0.2843	0.6018	0.6791	0.063*	
C8	0.4640 (2)	0.5017 (2)	0.5607 (2)	0.0427 (5)	
C3	0.1383 (3)	0.4844 (2)	0.12679 (19)	0.0427 (5)	
C1	0.6168 (4)	0.1027 (3)	0.0828 (3)	0.0689 (7)	
H1A	0.5056	0.1163	0.0916	0.103*	
H1B	0.6669	0.0753	0.0049	0.103*	
H1C	0.6613	0.1934	0.0978	0.103*	
C2	0.5666 (4)	0.0162 (4)	0.2865 (3)	0.0827 (9)	
H2A	0.6138	0.1018	0.3077	0.124*	
H2B	0.5807	−0.0678	0.3355	0.124*	
H2C	0.4559	0.0344	0.2961	0.124*	
N1	−0.0065 (2)	0.4450 (2)	0.1321 (2)	0.0606 (6)	
H1D	−0.0779	0.5115	0.1317	0.073*	
H1E	−0.0300	0.3526	0.1361	0.073*	
N2	0.1747 (3)	0.6261 (2)	0.12075 (19)	0.0590 (5)	
H2D	0.1034	0.6928	0.1203	0.071*	
H2E	0.2697	0.6517	0.1173	0.071*	
N3	0.2482 (2)	0.3819 (2)	0.12657 (18)	0.0556 (5)	
H3A	0.2247	0.2896	0.1299	0.067*	
H3B	0.3432	0.4077	0.1231	0.067*	
N4	0.6420 (3)	−0.0140 (2)	0.1648 (2)	0.0598 (6)	
H4A	0.7465	−0.0261	0.1558	0.072*	
H4B	0.6042	−0.0995	0.1478	0.072*	
C9	−0.0137 (2)	−0.0556 (2)	0.3530 (2)	0.0429 (5)	
C10	−0.0994 (3)	−0.1839 (2)	0.3832 (2)	0.0513 (6)	
H10	−0.1247	−0.2368	0.3256	0.062*	
C11	−0.1487 (3)	−0.2353 (3)	0.4988 (2)	0.0553 (6)	
H11	−0.2053	−0.3231	0.5174	0.066*	
C12	−0.1155 (3)	−0.1596 (2)	0.5852 (2)	0.0471 (5)	
H12	−0.1513	−0.1950	0.6622	0.057*	
C13	−0.0266 (2)	−0.0268 (2)	0.55895 (19)	0.0400 (5)	
S2	0.03040 (7)	0.01286 (6)	0.20605 (5)	0.04819 (17)	
O4	0.1966 (2)	0.0447 (2)	0.16779 (16)	0.0668 (5)	
O5	−0.0671 (2)	0.14568 (17)	0.20878 (16)	0.0590 (5)	
O6	−0.0188 (2)	−0.10150 (18)	0.14233 (15)	0.0602 (5)	

### Atomic displacement parameters (Å^2^)

**Table:**

	*U*^11^	*U*^22^	*U*^33^	*U*^12^	*U*^13^	*U*^23^
S1	0.0427 (3)	0.0350 (3)	0.0622 (4)	−0.0011 (2)	−0.0178 (3)	−0.0047 (2)
O1	0.0424 (9)	0.0513 (10)	0.0678 (11)	−0.0067 (7)	−0.0152 (8)	−0.0002 (8)
O2	0.0584 (10)	0.0448 (9)	0.0686 (11)	0.0028 (7)	−0.0273 (9)	0.0006 (8)
O3	0.0678 (11)	0.0400 (9)	0.0772 (12)	−0.0006 (8)	−0.0229 (9)	−0.0146 (8)
C4	0.0360 (11)	0.0324 (11)	0.0651 (15)	0.0022 (8)	−0.0140 (10)	−0.0035 (10)
C5	0.0471 (13)	0.0504 (14)	0.0751 (18)	0.0144 (11)	−0.0185 (12)	0.0007 (12)
C6	0.0512 (14)	0.0605 (16)	0.0755 (19)	0.0291 (12)	−0.0060 (13)	−0.0005 (13)
C7	0.0422 (12)	0.0445 (13)	0.0643 (16)	0.0123 (10)	−0.0045 (11)	−0.0032 (11)
C8	0.0332 (10)	0.0270 (10)	0.0672 (14)	0.0005 (8)	−0.0113 (9)	−0.0049 (9)
C3	0.0430 (12)	0.0413 (12)	0.0410 (12)	−0.0017 (9)	−0.0073 (9)	0.0038 (9)
C1	0.083 (2)	0.0507 (16)	0.0703 (19)	0.0003 (14)	−0.0156 (15)	−0.0022 (13)
C2	0.091 (2)	0.087 (2)	0.069 (2)	−0.0184 (18)	−0.0177 (17)	−0.0047 (16)
N1	0.0445 (11)	0.0447 (11)	0.0905 (16)	−0.0045 (9)	−0.0166 (11)	0.0133 (11)
N2	0.0546 (12)	0.0397 (11)	0.0831 (16)	−0.0032 (9)	−0.0198 (11)	0.0034 (10)
N3	0.0431 (11)	0.0402 (11)	0.0812 (15)	−0.0005 (8)	−0.0131 (10)	0.0006 (10)
N4	0.0527 (12)	0.0479 (12)	0.0803 (16)	0.0023 (9)	−0.0204 (11)	−0.0036 (10)
C9	0.0386 (11)	0.0312 (10)	0.0617 (14)	0.0016 (8)	−0.0181 (10)	−0.0046 (9)
C10	0.0539 (13)	0.0364 (12)	0.0693 (17)	−0.0070 (10)	−0.0249 (12)	−0.0084 (11)
C11	0.0559 (14)	0.0379 (12)	0.0763 (18)	−0.0165 (10)	−0.0238 (13)	−0.0006 (11)
C12	0.0428 (12)	0.0356 (11)	0.0636 (15)	−0.0065 (9)	−0.0151 (10)	−0.0003 (10)
C13	0.0296 (10)	0.0284 (10)	0.0645 (14)	0.0024 (8)	−0.0162 (9)	−0.0051 (9)
S2	0.0479 (3)	0.0357 (3)	0.0614 (4)	−0.0034 (2)	−0.0142 (3)	−0.0054 (2)
O4	0.0489 (10)	0.0736 (13)	0.0720 (12)	−0.0070 (9)	−0.0026 (8)	−0.0166 (10)
O5	0.0647 (11)	0.0373 (9)	0.0733 (12)	0.0020 (8)	−0.0168 (9)	0.0048 (8)
O6	0.0807 (12)	0.0417 (9)	0.0647 (11)	−0.0087 (8)	−0.0296 (9)	−0.0050 (8)

### Geometric parameters (Å, º)

**Table:**

S1—O1	1.4442 (16)	C2—H2C	0.9600
S1—O3	1.4448 (17)	N1—H1D	0.8600
S1—O2	1.4614 (17)	N1—H1E	0.8600
S1—C4	1.782 (3)	N2—H2D	0.8600
C4—C5	1.366 (3)	N2—H2E	0.8600
C4—C8^i^	1.436 (3)	N3—H3A	0.8600
C5—C6	1.392 (4)	N3—H3B	0.8600
C5—H5	0.9300	N4—H4A	0.9000
C6—C7	1.354 (4)	N4—H4B	0.9000
C6—H6	0.9300	C9—C10	1.375 (3)
C7—C8	1.421 (3)	C9—C13^ii^	1.437 (3)
C7—H7	0.9300	C9—S2	1.774 (2)
C8—C8^i^	1.421 (5)	C10—C11	1.391 (3)
C8—C4^i^	1.436 (3)	C10—H10	0.9300
C3—N1	1.312 (3)	C11—C12	1.362 (3)
C3—N2	1.320 (3)	C11—H11	0.9300
C3—N3	1.322 (3)	C12—C13	1.421 (3)
C1—N4	1.454 (3)	C12—H12	0.9300
C1—H1A	0.9600	C13—C13^ii^	1.422 (4)
C1—H1B	0.9600	C13—C9^ii^	1.437 (3)
C1—H1C	0.9600	S2—O4	1.4404 (18)
C2—N4	1.465 (4)	S2—O6	1.4496 (17)
C2—H2A	0.9600	S2—O5	1.4552 (17)
C2—H2B	0.9600		
			
O1—S1—O3	113.70 (11)	C3—N1—H1D	120.0
O1—S1—O2	111.13 (10)	C3—N1—H1E	120.0
O3—S1—O2	112.70 (11)	H1D—N1—H1E	120.0
O1—S1—C4	105.95 (10)	C3—N2—H2D	120.0
O3—S1—C4	107.12 (11)	C3—N2—H2E	120.0
O2—S1—C4	105.59 (10)	H2D—N2—H2E	120.0
C5—C4—C8^i^	120.0 (2)	C3—N3—H3A	120.0
C5—C4—S1	118.99 (19)	C3—N3—H3B	120.0
C8^i^—C4—S1	121.01 (16)	H3A—N3—H3B	120.0
C4—C5—C6	120.5 (2)	C1—N4—C2	113.6 (2)
C4—C5—H5	119.7	C1—N4—H4A	108.9
C6—C5—H5	119.7	C2—N4—H4A	108.9
C7—C6—C5	121.4 (2)	C1—N4—H4B	108.9
C7—C6—H6	119.3	C2—N4—H4B	108.9
C5—C6—H6	119.3	H4A—N4—H4B	107.7
C6—C7—C8	120.6 (2)	C10—C9—C13^ii^	120.2 (2)
C6—C7—H7	119.7	C10—C9—S2	118.36 (18)
C8—C7—H7	119.7	C13^ii^—C9—S2	121.21 (16)
C8^i^—C8—C7	118.6 (3)	C9—C10—C11	120.6 (2)
C8^i^—C8—C4^i^	118.8 (2)	C9—C10—H10	119.7
C7—C8—C4^i^	122.6 (2)	C11—C10—H10	119.7
N1—C3—N2	120.1 (2)	C12—C11—C10	121.2 (2)
N1—C3—N3	119.9 (2)	C12—C11—H11	119.4
N2—C3—N3	119.9 (2)	C10—C11—H11	119.4
N4—C1—H1A	109.5	C11—C12—C13	120.5 (2)
N4—C1—H1B	109.5	C11—C12—H12	119.7
H1A—C1—H1B	109.5	C13—C12—H12	119.7
N4—C1—H1C	109.5	C12—C13—C13^ii^	119.2 (2)
H1A—C1—H1C	109.5	C12—C13—C9^ii^	122.6 (2)
H1B—C1—H1C	109.5	C13^ii^—C13—C9^ii^	118.3 (2)
N4—C2—H2A	109.5	O4—S2—O6	113.80 (11)
N4—C2—H2B	109.5	O4—S2—O5	112.38 (11)
H2A—C2—H2B	109.5	O6—S2—O5	111.31 (11)
N4—C2—H2C	109.5	O4—S2—C9	108.35 (11)
H2A—C2—H2C	109.5	O6—S2—C9	105.83 (10)
H2B—C2—H2C	109.5	O5—S2—C9	104.46 (10)
			
O1—S1—C4—C5	119.8 (2)	C13^ii^—C9—C10—C11	0.3 (3)
O3—S1—C4—C5	−118.5 (2)	S2—C9—C10—C11	−174.47 (18)
O2—S1—C4—C5	1.8 (2)	C9—C10—C11—C12	0.9 (4)
O1—S1—C4—C8^i^	−58.72 (19)	C10—C11—C12—C13	−1.2 (4)
O3—S1—C4—C8^i^	62.98 (19)	C11—C12—C13—C13^ii^	0.4 (4)
O2—S1—C4—C8^i^	−176.68 (16)	C11—C12—C13—C9^ii^	−178.9 (2)
C8^i^—C4—C5—C6	2.1 (4)	C10—C9—S2—O4	−130.74 (19)
S1—C4—C5—C6	−176.5 (2)	C13^ii^—C9—S2—O4	54.60 (19)
C4—C5—C6—C7	−0.8 (4)	C10—C9—S2—O6	−8.3 (2)
C5—C6—C7—C8	−0.7 (4)	C13^ii^—C9—S2—O6	177.01 (16)
C6—C7—C8—C8^i^	0.8 (4)	C10—C9—S2—O5	109.27 (19)
C6—C7—C8—C4^i^	−178.7 (2)	C13^ii^—C9—S2—O5	−65.39 (18)

Symmetry codes: (i) −*x*+1, −*y*+1, −*z*+1; (ii) −*x*, −*y*, −*z*+1.

### Hydrogen-bond geometry (Å, º)

**Table:**

*D*—H···*A*	*D*—H	H···*A*	*D*···*A*	*D*—H···*A*
N1—H1*D*···O1^iii^	0.86	2.10	2.916 (3)	159
N1—H1*E*···O5	0.86	2.02	2.825 (3)	157
N2—H2*D*···O6^iv^	0.86	2.12	2.942 (3)	160
N2—H2*E*···O2	0.86	2.08	2.921 (3)	164
N3—H3*A*···O4	0.86	2.24	3.084 (3)	167
N3—H3*B*···O3	0.86	2.11	2.940 (3)	163
N4—H4*A*···O6^v^	0.90	2.12	3.011 (3)	168
N4—H4*A*···O5^v^	0.90	2.50	3.133 (3)	128
N4—H4*B*···O1^vi^	0.90	2.60	3.152 (3)	121
N4—H4*B*···O2^vi^	0.90	2.04	2.914 (3)	163

Symmetry codes: (iii) *x*−1, *y*, *z*; (iv) *x*, *y*+1, *z*; (v) *x*+1, *y*, *z*; (vi) *x*, *y*−1, *z*.
